# In vitro simulation research on the hoop stress of myocardial bridge - coronary artery

**DOI:** 10.1186/s13019-015-0261-6

**Published:** 2015-04-25

**Authors:** Hao Ding, Kun Shang, Hailian Lan, Yanan Lei, Lixing Sheng, Zhilin Liu, Yanjun Zeng

**Affiliations:** 1 Shanghai Medical Instrumentation College, Shanghai, 200093 China; 2 School of Medical Instrument and Food Engineering, University of Shanghai for Science and Technology, Shanghai, 200093 China; 3 Shanghai General Hospital, Shanghai, 200080 China; 4 Biomechanics & Medical Information Institute, Beijing University of Technology, No. 100 PingLeYuan, Beijing, 100022 China

**Keywords:** Mural coronary artery, Myocardial bridge, Hemodynamic, Hoop stress

## Abstract

**Aims:**

The aim of this study is to investigate how the myocardial bridge oppression affects the hoop stress of mural coronary artery.

**Methods:**

The “myocardial bridge – coronary artery” simulative device records the hoop stress which is changed by adjusting the external pressure of the simulated coronary artery and the oppression degree of the respectively.

**Results:**

Simulation experiment in vitro indicates that the abnormal hoop stress mainly occurs in the proximal end of mural coronary artery. As the oppression degree of myocardial bridge increases, the mean and the oscillatory value (maximum-minimum) of hoop stress in the proximal end increase markedly.

**Conclusions:**

The “myocardial bridge – coronary artery” simulation device can provide an experiment method of studying the hoop stress influence on mural coronary artery in vitro.

## Background

The muscle overlaying the mural coronary artery is termed a myocardial bridge (MB). To objectively evaluate clinical relevance of the MB, the haemodynamic behaviour of the mural coronary artery is of great significance. Previous studies, mostly considering clinical patients or experimental animals as subjects, have limitations. For example, haemodynamic parameters (MB width, degree of the mural coronary artery compression, blood pressure, heart rate, etc.) of research objects are stochastic, which makes it difficult to conduct systematic and comprehensive research. Also there have not been any clinically satisfactory methods to measure the blood flow of the mural coronary artery, which is the most important factor affecting myocardial perfusion state. Therefore, we lack sufficient knowledge about the link between MB and variation in blood flow of the mural coronary artery [1-5]. The myocardial bridge - mural coronary artery model developed in this paper is a complement to clinical research and results from experimental animals.

Hoop stress is an important hemodynamic factor. Studies have shown that cells from vessel wall are simultaneously subjected to both shear stresses and hoop stresses caused by blood flow. Cells from vessel wall can identify these mechanical stimuli and transfer these signals into the inside of cells, leading to changes in cellular morphology, function and gene expression. It is thought that the biological responses of vessel wall cells to mechanical stimuli (or changes in mechanical environment) are closely associated with hypertension, atherosclerosis and vascular remodeling such as restenosis after vascular transplantation [6]. Therefore, investigating hoop stress of myocardial bridge and mural coronary artery is conducive to explore the correlation between atherosclerosis and hemodynamic environment. It also has the potential clinical implication for pathogenesis and treatment.

A patient needs treatment when mural coronary artery (MC )stenosis is over 50%. Sometimes the effect of drug therapy is poor for some MB patients and a part of MB patients even cannot tolerate drug therapy, so percutaneous transluminal coronary angioplasty or stent implantation is usually used for them. Coronary artery stent implantation not only can correct the abnormal MC hemodynamics, but also can make the MC blood flow return to normal values. However, 10% - 50% of the patients may develop stent restenosis after performing coronary artery stent implantation and even more seriously, the coronary artery injury may occur during operation or post-operation [7-9].

The hoop stress, referred as the stress along the tangential direction of the arterial wall’s cross section, is one of the important factors in vascular plaque rupture [10,11]. Therefore, the research on MC hoop stress distribution can help us to prevent the coronary artery injury during or after stent implantation.

Once the value of hoop stress is determined, the tensile stress in the strain chamber system can be used to simulate the hoop stress [12]. However, the strain chamber system can only simulate the normal blood vessel in vitro being subjected to the hoop stress. It cannot simulate the hoop stress environment where the MB develops around the coronary artery [13,14].

Here we design a new device which is able to simulate the MB oppressing the coronary artery, acquire the parameters related to the hoop stress, and provide repeatable and controllable experimental conditions.

## Methods

### The structure and function of the device

**Figure 1:**
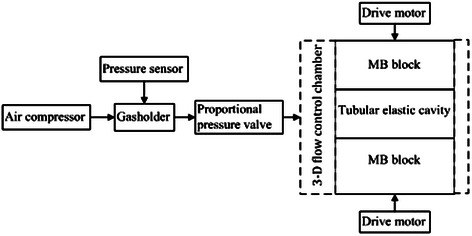
Block diagram of the “myocardial bridge- coronary artery” simulative device structure.

Figure 1 is the block diagram of the “myocardial bridge - coronary artery” simulative device. Air is pressurized by a compressor and then delivered to the air tank to make the pressure stable. The pressure sensor is placed at the top of the gasholder to measure the internal pressure. Then gas flows through a proportional pressure valve, and eventually enters into the closed 3-D flow control chamber, where the pressurized gas can provide external pressure for tubular elastic lumen, thus change the external wall pressure, and in turn regulate the hoop stress. When the internal pressure of the air tank rises to 30kPa, the air compressor will stop inflating. When the internal pressure of the 3-D flow control chamber is lower than the set value, the gas stored in the air tank will continuously flow into the 3-D flow control chamber through the proportional pressure valve. When the internal pressure of the 3-D flow control chamber is higher than the set value, the air tank will stop supplying the gas and the proportional pressure valve will release the gas so as to reduce the pressure. The drive motor can drive the MB blocks to oppress the tubular elastic lumen.

**Figure 2:**
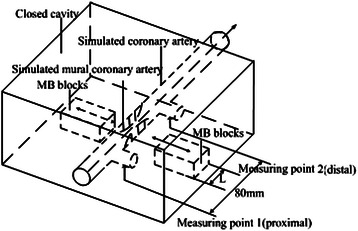
The map of three-dimensional flow control chamber.

Figure 2 is the diagram of the 3-D flow control chamber. The chamber is a closed Lucite cube and the internal situation can be observed clearly. The size of the internal chamber is 125mm × 110mm × 55mm (length × width × height). The volume of the 3-D flow control chamber is much larger than that of the simulated coronary artery. The device can simulate the coronary artery with a inner diameter of 4 ~ 5mm and length of 80mm. Measuring point 1 is set at the inlet of the chamber to measure the proximal pressure while measuring point 2 is located at the outlet to measure the distal pressure. The MB blocks are set at both radial sides of the simulated MC. The blocks, which are driven by a motor, undergo the straight reciprocating motion along the guide rail according to the preset depth to oppress the simulated MC.

The device can perform bilateral or unilateral compression, and different sized blocks can be chosen to get different compression width.

### Theoretical basis

The hoop stress of the circular tube can be obtained through the following equation [15]:

1 $$ {\sigma}_{\theta }=\frac{\frac{{r_0}^2}{r^2}+1}{\frac{{r_0}^2}{{r_i}^2}-1}{q}_1-\frac{1+\frac{{r^2}_i}{r^2}}{1-\frac{{r_i}^2}{{r_0}^2}}{q}_2, $$

where *σ*_*θ*_ is the hoop stress of the wall, *q*_1_is the internal wall pressure, *q*_2_ is the external wall pressure, *r*_0_ is the external radius with the load, *r*_*i*_ is the internal radius with the load, and *r* is the radius at any point with the load.

To calculate the internal wall hoop stress, we set *r* to be *r*_*i*_. In fact, the radius *r*_0_ and *r*_*i*_ of the elastic tube are influenced by the pressure wave. Therefore, to obtain the geometrical and mechanical characteristics of the silicone tube flow chamber under pulsatile conditions, we use the pressure sensor and the parallel light camera to measure the pressure wave and the external radius wave of the silicone tube flow chamber, respectively. By fitting the pressure wave and the external radius wave, we get the following equation.

2 $$ {d}_0=a+b\times p+c\times {p}^2-d\times {p}^3+e\times {p}^4, $$

where *d*_*0*_ is the external diameter of the loading silicone tube, that is, *d*_*0*_ = *2r*_*0*_. *p* is the internal-external pressure difference of silicone tube, that is, *p* = *q*_*1*_- *q*_*2*_; and the other parameters as follow:

$$ \mathrm{a}=4.91\kern0.5em \mathrm{b}=3.17\times {10}^{-3}\kern0.5em \mathrm{c}=1.18\times {10}^{-5}\kern0.5em \mathrm{d}=4.81\times {10}^{-7}\kern0.5em \mathrm{e}=2.39\times {10}^{-9} $$

*R*_*i*_ denotes the initial internal radius of the silicone tube without the load, while *R*_0_ denotes the initial external radius of the silicone tube without the load. Due to the incompressible characteristic of the silicone tube [13], we have

3 $$ {\lambda}_z\pi \left({r_0}^2(t)-{r}_i^2(t)\right)=\pi \left({R_0}^2-{R_i}^2\right). $$

Where *λ*_*z*_ is the longitudinal stretch ratio. Thus, the relationship between the internal and external radius of the tube with the load is

4 $$ {r}_i(t)=f\left({r}_0(t)\right)=\sqrt{r_0{(t)}^2-\frac{1}{\lambda_z}\left({R_0}^2-{R_i}^2\right)}. $$

Since *λ*_*z*_ = 1.2, *R*_0_ =2.465 mm, *R*_*i*_ =2.165 mm,we have

5 $$ {r}_i(t)=f\left({r}_0(t)\right)=\sqrt{{r_0}^2(t)-\frac{1}{1.2}\left({2.465}^2-{2.165}^2\right)}=\sqrt{{r_0}^2(t)-1.1575}. $$

Then, the hoop stress of the silicone tube flow chamber system under pulsatile conditions can be calculated by

6 $$ {\sigma}_{\theta }=\frac{2{r_0}^2-1.1575}{1.1575}{q}_1-\frac{2{r_0}^2}{1.1575}{q}_2. $$

According to the equation (2) and equation (6), we can calculate hoop stress by internal pressure q_1_ and external pressure q_2_ which are measured through pressure sensor.

### Experimental design

According to the clinical parameters of healthy adult humans [16], we set that the systolic blood pressure of coronary artery is 120 mmHg, the diastolic pressure is 80 mmHg, the mean flow is 205 ml/min, and the heart rate is 60 bmp. During systole, the myocardial bridge oppresses the mural coronary artery [17-20]. Therefore, in the experiment, MB oppresses MC at the same frequency as heart rate during systole, and the maximal output pressure and the maximal compression of MB keep synchronous. The fluid in the tube is a mixture of low molecular dextran and saline with a ratio of 3:1, whose viscosity is 3.8 × 10^−3^ Pa·S [21].

The experimental protocol was as follows.

1) Compare the MC proximal and distal pressure waves of clinical MB patients with the results of the simulative device; 2) In the case that the external pressure of coronary artery is constant, change MB compression level to observe the changes in the proximal and distal hoop stress; 3) In absence of MB compression change the external pressure of the tube to observe the changes in the proximal and distal hoop stress.

## Results

### Pressure wave

**Figure 3:**
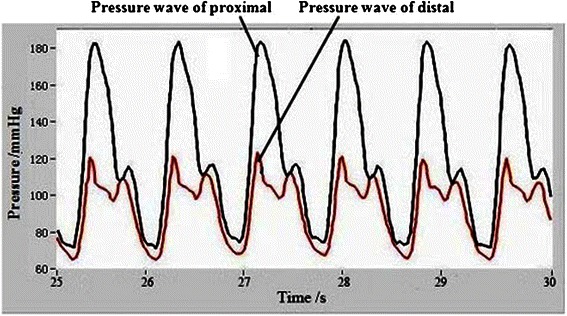
The proximal and distal pressure wave of mural coronary artery in the simulative device.

Figure 3 shows the pressure wave of the simulative device when MB compression level is 100%. The simulation results are in agreement with clinical MC pressure waves, showing “water hammer” phenomenon [22,23].

### Proximal and distal hoop stress when MB oppresses MC

**Figure 4:**
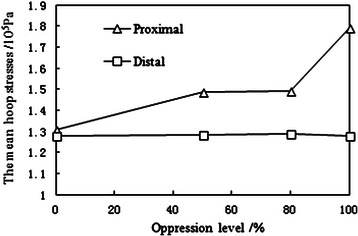
The mean hoop stresses of the simulated mural coronary artery.

**Figure 5:**
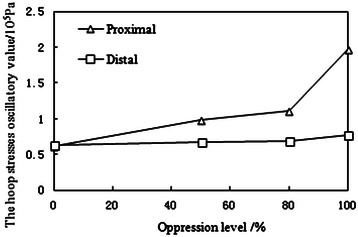
The hoop stresses oscillatory value of the simulated mural coronary artery.

Under normal atmospheric pressure, the simulative MB oppresses MC at different levels. Using the experimental data, we plot the curves of the mean proximal and distal hoop stresses with different oppression levels shown in Figure 4. As the oppression level increases, the mean proximal hoop stress increases significantly, while the mean distal hoop stress remains unchanged. The oscillatory value of the proximal hoop stress (maximum-minimum) is markedly higher than that of the distal hoop stress. Moreover, the increase in oppression level leads to the increase in the oscillatory value of proximal hoop stress. (See Figure 5)

### Independent regulation of hoop stress

**Figure 6:**
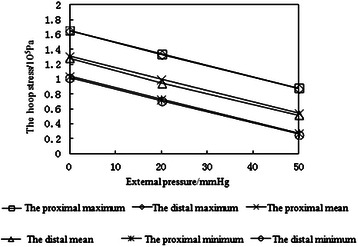
The hoop stresses changing with external pressure of the simulated mural coronary artery.

As shown in Figure 6, the hoop stress in the inner wall of simulated mural coronary artery depends on the external pressure exerted on the silicone pipe.

The simulative device can maintain constant internal pressure q_1_, flow, and other parameters stable. One can change the external pressure q_2_ to adjust the value of internal hoop stress. The larger q_2_ is, the less the hoop stress is.

## Discussion

The simulation experiment results show that the oscillation of hoop stress of mural coronary artery in the proximal end is very severe. With the increasing oppression of myocardial bridge on mural coronary artery, the average value and fluctuating value of hoop stress of mural coronary artery in the proximal end increase significantly, while those values in the distal end remain unchanged basically.

1. The average value of stress

The greater oppression of myocardial bridge on mural coronary artery, the greater normal stress and hoop stress of mural coronary artery in the proximal end. The normal stress increases gradually because the blood flow kinetic energy is transformed into the pressure energy in the moment of mural coronary artery oppressed by myocardial bridge. The hoop stress in the proximal end increase accordingly due to the hoop stress is proportional to the normal stress.

(2) The oscillatory value of stress

With the increasing oppression of myocardial bridge on mural coronary artery, the fluctuating (degree) of normal stress and hoop stress in the proximal end becomes more intense than that in the distal end. The normal blood flow is blocked because of the instantaneous myocardial bridge oppression in the proximal end of mural coronary artery, which causes “water hammer phenomenon”.

The local “tensile stress concentration” phenomenon happens when average hoop stress of mural coronary artery in the proximal end increases. It will result in higher hoop stress in the proximal local area than that in surrounding area. On the other hand, the increasing oscillatory value of the hoop stress will make the blood vessels under higher fatigue load. Since the fatigue load is so sensitive to stress concentration, it can cause fatigue damage to the blood vessels more easily. That is the main reason of the AS multifocal distribution [24].

In fact, there are four oppression degrees of MC caused by MB, including 0%, 50%, 80% and 100%. With the oppression degree increasing, the proximal pressure increases gradually, but the distal pressure remains unchanged. It only shows the typical pressure wave under 100% oppression in this paper because its representativeness. According to the equation (6), the proximal and distal hoop stress of MC is a function about internal pressure q_1_ and external pressure q_2_ of circular tube, So the hoop stress value can be adjusted by changing external pressure q_2_ in the condition of internal pressure q_1_ unchanged.

The main purpose of this device is not to imitate a certain clinical symptoms, but to provide an experimental environment. The change of hoop stress of myocardial bridge - coronary artery can be explored in some specific physiological conditions, such as blood pressure, blood flow and heart rate etc. As to any situation that the change of external pressure in the blood vessel wall clinically whether exist or not, there isn’t sufficient relative literature report yet.The hoop stress can be calculated by equation (6) approximately because the change of internal diameter of the proximal and distal MC is so negligible that it can be ignored when the MC oppressed by MB. But the intense internal diameter deformation occurred in the lumen of MC, so the equation (6) is not suitable for calculating hoop stress. Meanwhile, the method of acquiring hoop stress of MC lumen needs to be further studied in the future work.

## Conclusions

The hoop stress plays an important role in the process of the blood vessels reconstruction, while some researchers who research on blood flow dynamics at home and abroad focused on shear stress mostly at present, but the research on (about) hoop stress is relatively rare.

The influence of the abnormal MC proximal hoop stress induced by MB on the coronary artery disease has not been reported. Meanwhile, whether curative effect of interventional therapy about severe AS in the proximal MB will be impacted by MB or not is unclear [25-27].

Some physiological parameters, such as blood pressure and blood flow etc., will be affected by myocardial bridge - coronary artery. Currently, it is hard to research on clinical symptoms because of some changes of single and specific hemodynamic parameters.The relationship between hypertension and atherosclerosis is the mutual cause-effect [28-30]. As a result, the increasing blood pressure caused by MB in the proximal is closely related with atherosclerotic lesions.

The hoop stresses of MC in the proximal and distal end is simulated and calculated through simulation device in this paper. The results indicate that the proximal pressure is much higher than the distal pressure. Consequently, the abnormal hoop stress mainly occurs in the proximal end of MC. As the oppression degree of myocardial bridge increases, the average and the oscillatory value (maximum-minimum) of in the proximal end increase markedly. The increasing average value of hoop stress will result in higher risk of the proximal MC injury. Meanwhile, as the oscillatory value increases, the MC in the proximal end is in the long-term fatigue load situation, which will increase the probability of the MC injury in the proximal.

## Abbreviations

MB: Myocardial bridge
MC: Mural coronary artery
